# A Preprocessing Pipeline for Pupillometry Signal from Multimodal iMotion Data

**DOI:** 10.3390/s25154737

**Published:** 2025-07-31

**Authors:** Jingxiang Ong, Wenjing He, Princess Maglanque, Xianta Jiang, Lawrence M. Gillman, Ashley Vergis, Krista Hardy

**Affiliations:** 1 Department of Surgery, University of Manitoba Max Rady College of Medicine, Winnipeg, MB R3E 0W2, Canada; ongj2@myumanitoba.ca (J.O.); maglanpm@myumanitoba.ca (P.M.); lawrence.gillman@umanitoba.ca (L.M.G.); avergis@sbgh.mb.ca (A.V.); khardy@sbgh.mb.ca (K.H.); 2 Department of Computer Science, Memorial University of Newfoundland, St. John’s, NL A1B 3X7, Canada; xiantaj@mun.ca

**Keywords:** pupillometry, multimodal data, preprocessing, median absolute deviation (MAD), moving average (MA) filter, piecewise cubic hermite interpolatingpolynomial (PCHIP), linear regression

## Abstract

Pupillometry is commonly used to evaluate cognitive effort, attention, and facial expression response, offering valuable insights into human performance. The combination of eye tracking and facial expression data under the iMotions platform provides great opportunities for multimodal research. However, there is a lack of standardized pipelines for managing pupillometry data on a multimodal platform. Preprocessing pupil data in multimodal platforms poses challenges like timestamp misalignment, missing data, and inconsistencies across multiple data sources. To address these challenges, the authors introduced a systematic preprocessing pipeline for pupil diameter measurements collected using iMotions 10 (version 10.1.38911.4) during an endoscopy simulation task. The pipeline involves artifact removal, outlier detection using advanced methods such as the Median Absolute Deviation (MAD) and Moving Average (MA) algorithm filtering, interpolation of missing data using the Piecewise Cubic Hermite Interpolating Polynomial (PCHIP), and mean pupil diameter calculation through linear regression, as well as normalization of mean pupil diameter and integration of the pupil diameter dataset with facial expression data. By following these steps, the pipeline enhances data quality, reduces noise, and facilitates the seamless integration of pupillometry other multimodal datasets. In conclusion, this pipeline provides a detailed and organized preprocessing method that improves data reliability while preserving important information for further analysis.

## 1. Introduction

Pupillometry is the measurement of changes in pupil diameter, a physiological process that provides important insights into human cognition, emotional states, and stress levels [1,2]. Recent research shows that, beyond humans, pupil size in monkeys is also a sensitive marker of emotional arousal and social relevance, especially in response to conspecific facial expressions [3]. Pupil diameter is influenced by several factors, such as light intensity, mental effort, emotional arousal, and cognitive load [4]. Due to its strong correlation with cognitive workload, pupillometry has become a popular and objective physiological measure for evaluating attentional demands and mental effort during complex tasks [5,6,7,8].

With technological advancements, multimodal research has gained more attention as it enables researchers to combine pupillometry with other physiological and behavioral measures to enhance the understanding of human responses. The iMotions platform facilitates this integration by synchronizing pupillometry with data streams such as video for facial expression, Electroencephalogram (EEG), and other channels of data to allow for a comprehensive analysis of cognitive and emotional states [9]. Despite these advancements, preprocessing pupillometry data becomes more challenging in a multimodal context, and there is no standardized pipeline for processing pupillometry data. Existing studies primarily focus on preprocessing single-channel eye-tracking data [10], where preprocessing usually involves managing artifacts such as blinks, tracking errors, and missing data [11].

One major challenge in multimodal research is synchronizing multiple data streams operating at different sampling rates, which complicates data integration. Additionally, timestamp misalignment that occurs after interpolation often requires further adjustments to maintain consistency across datasets. Blinks and occlusions introduce missing values and sudden changes in pupil diameter [12], which can undermine the reliability of the analysis if not handled carefully. Therefore, removing these artifacts is critical to ensure data integrity. Furthermore, merging pupillometry data with other physiological signals, such as facial expression analysis, requires precise alignment to ensure appropriate interpretation of cognitive workload and facial expression responses. Without systematic preprocessing techniques, these challenges may result in inconsistencies and inaccuracies in the analysis of pupillary dynamics within multimodal research settings.

Our preprocessing pipeline builds upon the key steps described by Kret and Sjak-Shie, whose methods we reference in our manuscript [11]. We have adopted and improved these methods to better address the challenges of multimodal pupillometry data and to enhance the handling of pupil size signals. The authors acknowledge the foundational role their work played in the development of our pipeline.

To build upon this foundation, this study aims to address these gaps by introducing a systematic preprocessing pipeline tailored for multimodal pupillometry data collected using the iMotions platform. The proposed pipeline generates a clean, continuous dataset of pupil diameter measurements from the raw eye-tracking data acquired with iMotions 10. To generate a smooth and reliable representation of pupil diameter, the preprocessing pipeline incorporates steps such as artifact removal, interpolation of missing data, normalization, and integration of facial expression data. By offering a structured and practical approach to handling multimodal pupillometry data, this study provides researchers with a practical pipeline for improving data quality and ensuring accurate analysis of cognitive workload, task performance, and facial expression responses in experimental research.

## 2. Materials and Methods

### 2.1. Participant and Experimental Setup

**Figure 1:**
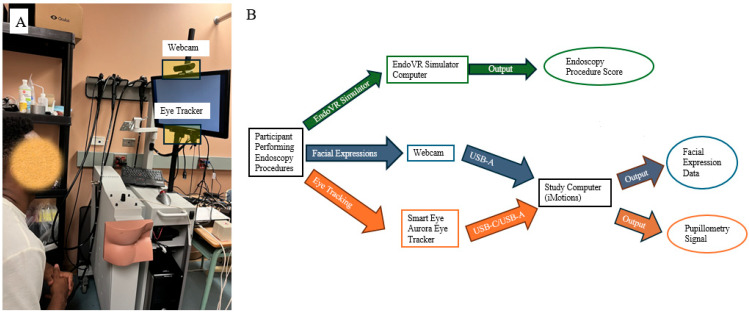
(**A**) Endoscopic simulator setup; (**B**) schematic diagram of data acquisition.

The participant, a novice in endoscopy who had completed fewer than ten endoscopic procedures, was instructed to perform endoscopic procedures with the CAE EndoVR simulator (Figure 1A. CAE Healthcare Inc, Montreal, QC, Canada). The endoscopic VR simulator includes a display screen with a webcam on top of the screen to record user interactions and an Smart Eye Aurora Eye Tracker mounted below the screen to monitor gaze patterns (Figure 1A). Figure 1B presents a schematic diagram illustrating how facial expressions and eye tracking data are captured by the webcam and the Smart Eye Aurora Eye Tracker (Smart Eye Inc, Gothenburg, Sweden) during the simulated endoscopy procedures.

Before the trial, the participant was given three minutes to practice in order to familiarize themselves with the simulation and the scenario. Once the simulation began, no specific feedback was provided regarding their performance. During the procedure, pupil diameter data was collected to examine the relationship between task performance, facial expression responses, and pupil dilation.

### 2.2. Pupillometry Data Preprocessing and Integration Workflow

**Figure 2:**
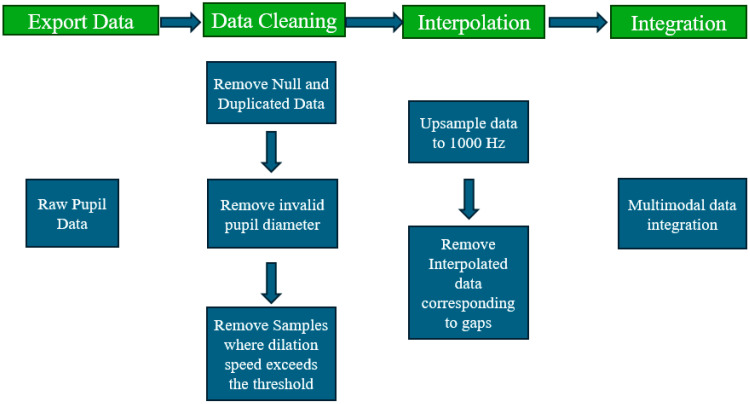
Pupillometry signal preprocessing and multimodal data integration process. The flowchart outlines the pupillometry signal preprocessing and multimodal data integration process that starting with raw pupil data.

This study is part of a larger project involving more than 20 participants aimed at analyzing endoscopy performance by examining pupillometry and facial expression response data. For this paper, a single participant was selected to demonstrate the preprocessing methods. The overall process of pupillometry signal preprocessing and multimodal data integration involves raw pupil data, data cleaning, interpolation, and integration steps (Figure 2).

The sequence of steps includes data cleaning, interpolation of missing values, upsampling and integration with other modalities [11,13]. These steps are established best practices for eye-tracking research and are widely recommended in the literature to address common challenges such as noise, missing data, and synchronization issues, and to ensure data quality and reliability for subsequent analysis. By following this workflow, our approach supports robust integration of pupillometry data with other multimodal datasets, aligning with current standards in the field.

### 2.3. Determine Relevant Timestamps from Video Annotation

Video annotation was used to identify specific timestamps or behavioral markers within the recorded procedures, serving as an important tool for decomposing a procedure into meaningful subtasks. This process can be performed under the iMotions software platform (iMotion, Copenhagen, Denmark) by adding landmarks to denote key events in the procedure.

To define the start and end points of the procedure, the first and last frame corresponded to the moment of scope insertion and scope withdrawal, respectively. This step was essential for analyzing the participant’s pupillary response during task execution and assessing its relationship with task performance and facial expressions during the endoscopic simulation [14].

### 2.4. Raw Multimodal Data from iMotions

The raw data was exported from iMotions 10 (version 10.1.38911.4), which included affective metrics such as facial expressions, pupil diameter measurements, and other eye-tracking parameters. This data formed the basis for examining the cognitive and facial expression dynamics of endoscopic performance.

Affective metrics and pupil diameter measurements were recorded at their sampling rates, as determined by the default settings of the iMotions and neon glasses software. Specifically, affective metrics were sampled at 30 Hz, while pupil diameter measurements were sampled at 120 Hz. As a result, each modality possesses its own set of recorded timestamps, leading to inconsistent and misaligned timestamps across the datasets.

#### Timestamp Misalignment in Raw Multimodal Data

Table 1 shows that some rows contain only facial expression data, while others contain only pupil data. Such timestamp misalignment is a common challenge in multimodal time series analysis, as differences in sampling rates and device-specific timing can result in incomplete temporal overlap between modalities.

### 2.5. Filtering Invalid and Null Pupil Diameter Data

**Figure 3:**
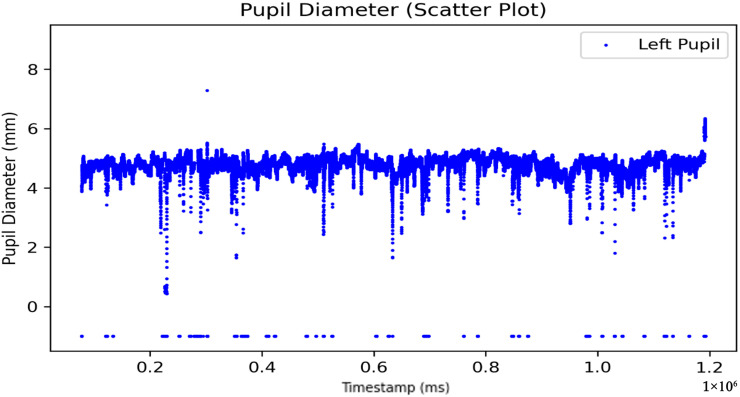
Unprocessed left eye pupil diameter data over time (ms).

To ensure accurate preprocessing, only the timestamp and pupil diameter variables were retained. Rows without pupil diameter data were removed to address missing data issues. Additionally, duplicate timestamps that might have been caused by software errors were also removed. As measurements were taken for both eyes, each eye was processed independently to avoid the accidental removal of valid data when filtering out invalid values. For example, unprocessed data of the left eye pupil are shown in Figure 3. This systematic approach ensures the accuracy and reliability of the dataset.

**Figure 4:**
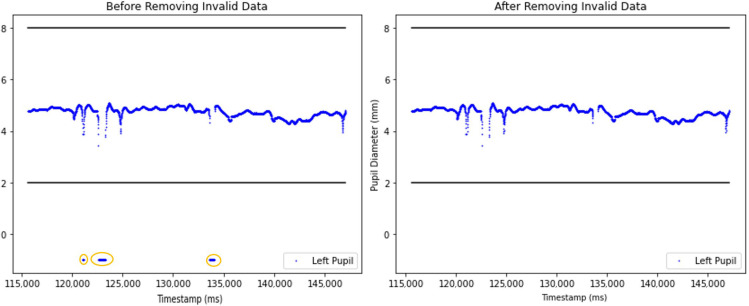
Comparison of unprocessed and processed pupil diameter data after invalid sample removal. The **left** graph illustrates the unprocessed dataset, which includes invalid pupil diameter samples below 2 mm. The circled area highlights pupil diamater data below 2. The **right** graph presents the dataset after removing invalid pupil diameter samples.

At the beginning of the preprocessing, null data and invalid pupil diameter samples were removed (Figure 4). Invalid pupil diameters were classified as diameter values falling outside the typical range of 2 mm to 8 mm, which often happened because of eye blinks, tracking system detection errors, or head movement [15].

### 2.6. Outlier Detection Based on Dilation Speed and Median Absolute Deviation

To identify and remove unrealistic changes in pupil diameter, a dilation speed outlier detection was used. Samples showing significant absolute changes in comparison to their adjacent samples were classified as dilation speed outliers. Each pupil diameter measurement $d\left[i\right]$ was paired with its corresponding timestamp t[i]. The normalized dilation speed metric $d^{\prime }\left[i\right]\;$ was computed to measure the maximum absolute change in pupil diameter in comparison to their adjacent samples. The formula below was used to calculate normalized dilation speed metric [16]

(1) $$d^{\prime }\left[i\right]=\max\left.\left(\left|\frac{d\left[i\right]-d\left[i-1\right]}{t\left[i\right]-t\left[i-1\right]}\right|\right.,\left|\frac{d\left[i+1\right]-d\left[i\right]}{t\left[i+1\right]-t\left[i\right]}\right|\right)$$

This method ensured outliers were removed from the dataset by simplifying the identification of pupil diameter changes that were disproportionate to corresponding time interval. To detect dilation speed outliers, a threshold was set using the median absolute deviation (MAD) of the dilation speed series. The MAD, which is the median of the absolute differences between each dilation speed measurement $d^{\prime }\left[i\right]\;$ and the median of the series ($median(d^{\prime }\left[i\right]))$, was calculated in the first stage. The MAD formula is given as:

(2) $$MAD=median(|d^{\prime }\left[i\right]-median(d^{\prime })|)$$

Next, the threshold for outlier detection was calculated by adding a multiple of the MAD to the median of the dilation speeds series. The threshold formula is expressed as:

(3) $$Threshold=median\left(d^{\prime }\right)+n\;*\;MAD$$

In this formula, n represents a scaling factor that determines the sensitivity of the outlier detection process. In this study, n was set to 20. Outlier samples were identified and removed if dilation speeds exceeded the threshold.

**Figure 5:**
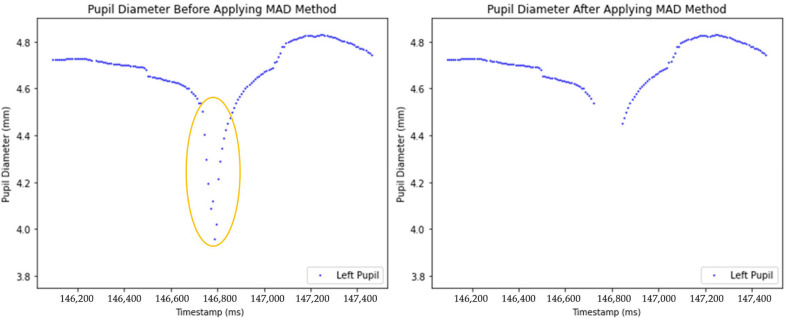
Comparison of unprocessed and processed pupil diameter data after anomaly removal.

Two plots comparing pupil diameter measurements before (left) and after (right) the application of outlier removal techniques are presented in Figure 5. The blue dots represent pupil diameter measurements, while the circled area highlights the sudden decrease in pupil diameter. Such irregularities likely happened due to blinking, tracking errors, sudden obstructions or head movement. After outlier removal, the right graph (Figure 5) presents a cleaner representation of pupil diameter changes.

### 2.7. Refining Local Trends with Moving Average-Based Outlier Detection

The previous outlier detection method compares all data points to the median, which is effective to identifying global outliers, particularly extreme values that deviate significantly from the overall distribution. However, this method may fail to detect local outliers with small deviations from the overall distribution. To address this, a moving average (MA) algorithms method was implemented to enhance sensitivity to local trends.

The Moving Average method smooths the pupil diameter signal by calculating the mean of consecutive data points within a window. This approach prioritizes local patterns, where smaller window sizes capture short-term fluctuations, and larger windows produce smoother trends. The MA at index i is computed as:

(4) $$MA\left[i\right]=\frac{x\left[i\right]+x\left[i-1\right]+\cdots +x\left[i-k+1\right]}{k}$$

where k represents the window size and $x\left[i\right]$ is the pupil diameter at timestamp i [17]. In this study, given that the sampling rate of pupil diameter measurement is 120 Hz, which is 8.33 milliseconds (ms) per frame and the typical blink durations range between 150 and 400 ms [18], the window size was chosen to minimize the influence of partial eyelid occlusions. Based on these parameters, an appropriate window size should be at least 18 to 48 samples, and a window size of 23 samples was used based on participant data.

The absolute deviation of each pupil diameter $d\left[i\right]\;$ measurement from its local MA trend was calculated using the following formula:

(5) $$d\left[i\right]=\left|x\left[i\right]-MA\left[i\right]\right|$$

This deviation quantifies the extent to which each data point deviates from the localized trend, enabling the identification of potential anomalies.

To identify outliers, a threshold was defined using the median of the deviation series. The threshold is expressed as:

(6) $$Threshold=n\;*\;median\left(d\right)$$

where n represents a scaling factor that determines the sensitivity of the outlier detection process. In this study, n was set to 18. Pupil diameters were classified as outliers and removed if their deviations exceeded this threshold [16].

This method effectively captures sudden spikes that deviate from nearby trends, often caused by partial eyelid occlusions [19]. By maintaining sensitivity to temporal dynamics, this approach offers a robust solution for detecting subtle local anomalies while preserving the integrity of the overall dataset.

**Figure 6:**
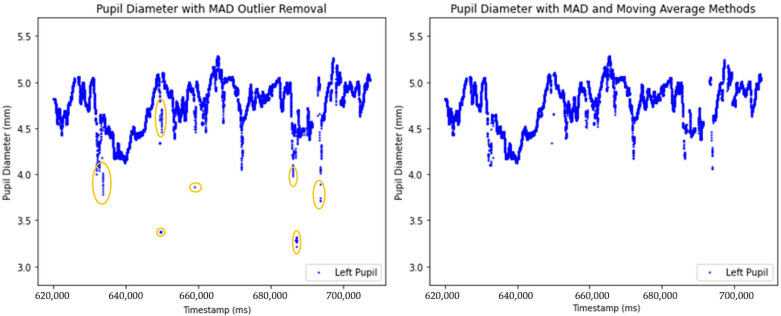
Comparison of unprocessed and processed pupil diameter data after anomaly removal using MAD and MA methods. The circled area highlights pupil diameter values identified as local outlier.

The effects of two outlier removal methods on pupil diameter data are illustrated in Figure 6. The left plot presents the dataset after applying the Median Absolute Deviation (MAD) method to detect outliers, which effectively removes global outliers. The right plot demonstrates the combined approach of MAD and a Moving Average (MA) to detect outliers, smoothing local fluctuations, and removes global outliers.

### 2.8. Interpolation and Upsampling of Pupil Diameter Data Using PCHIP

After removing outliers, interpolation was applied to create a smooth and high-resolution dataset. The initial eye-tracking data was recorded at 120 Hz and was upsampled to a 1000 Hz time series using Piecewise Cubic Hermite Interpolating Polynomial (PCHIP) [11,20] to form a smooth and continuous pupil size time series. Dan et al. (2020) systematically evaluated six interpolation methods including Previous Neighbor, Linear, Cubic Spline, Akima, Makima, and PCHIP on real pupil diameter recordings. Their results demonstrated that the most accurate and physiologically plausible reconstructions were obtained using Akima, Makima, and PCHIP because they consistently produced the smallest deviations from the true signal and best preserved the underlying trend of the data [20]. In our analysis, we compared these methods using Root Mean Squared Error (RMSE), which measures average error, and the Maximum Error, which captures the largest deviation. All three methods produced very similar, low error values, confirming their comparable accuracy (PCHIP: RMSE = 0.0033, Max Error = 0.2188; Akima: RMSE = 0.0032, Max Error = 0.2239; Makima: RMSE = 0.0032, Max Error = 0.2277). PCHIP was chosen for the study because it strictly preserves the shape of the data and minimizes unwanted oscillations, which is visually evident in our plots and aligns with our team’s preference for physiologically realistic results (Supplementary Figures S1–S3). All code and error metrics are included in our analysis pipeline for transparency. PCHIP is a piecewise interpolation technique designed to construct smooth curves through data points while preserving the shape and monotonicity of the original data, and overshooting issues [21]. Interpolating to 1000 Hz helps simplify time-series alignment and analysis. It creates a common, high-resolution time base, allowing all datasets, regardless of their original sampling rates, to be synchronized accurately to the millisecond. This is particularly important for multimodal integration with physiological signals such as EEG or ECG, which are often sampled at high frequencies [22,23,24,25].

Additionally, the interpolation process addresses missing values in the pupil data caused by blinks, looking away, or tracker occlusion by reconstructing a continuous signal. The high-resolution time grid also provides critical flexibility for subsequent downsampling to match time alignment of the facial expression datasets, which were recorded at 30 Hz. This ensures that the data can be adapted to different analytic needs without repeated interpolation or the risk of misalignment. Besides that, not all modalities are suitable for high-frequency interpolation. For example, facial expression data based on facial muscle analysis are typically recorded at lower rates and may not support meaningful interpolation to high resolutions due to different facial expressions have different measurement. There are no universal interpolation methods for all facial expressions, applying interpolation can make data overly complicated and highly inaccurate, resembling artificial data.

**Figure 7:**
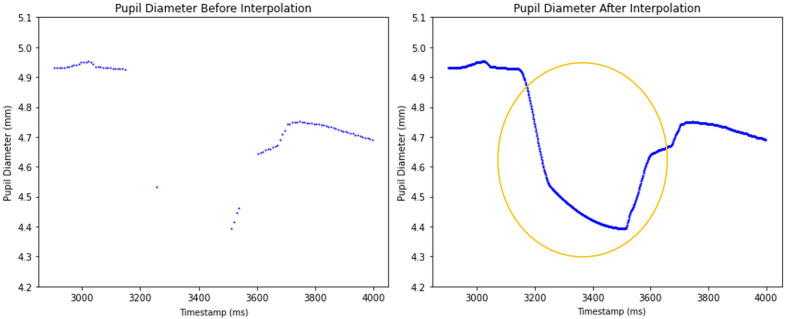
Comparison of unprocessed and processed pupil diameter data after applying PCHIP interpolation. The circled area highlights the region where PCHIP has interpolated the missing pupil diameter data.

The effect of the PCHIP interpolation method is illustrated in Figure 7. The left graph displays the unprocessed pupil diameter data with gaps due to missing values, while the right graph presents the dataset after PCHIP interpolation has been applied. The interpolated dataset effectively fills these gaps and reconstructing a smooth and continuous pupil size signal at the higher 1000 Hz sampling rate.

Next, the interpolated data points corresponding to gaps in the original dataset that exceeded 250 milliseconds were removed to maintain data integrity and prevent artificial signal distortion [11].

### 2.9. Binocular Data Alignment and Mean Pupil Diameter Estimation

To ensure alignment between left and right eye measurements following interpolation and artifact removal, all timestamps were rounded, and the datasets were integrated using an outer join. Generating an accurate mean pupil diameter from binocular eye-tracking data involves several challenges, particularly when measurements are missing for one eye at specific timestamps since pupil diameters are recorded independently for each eye, leading to incomplete data at certain points.

**Figure 8:**
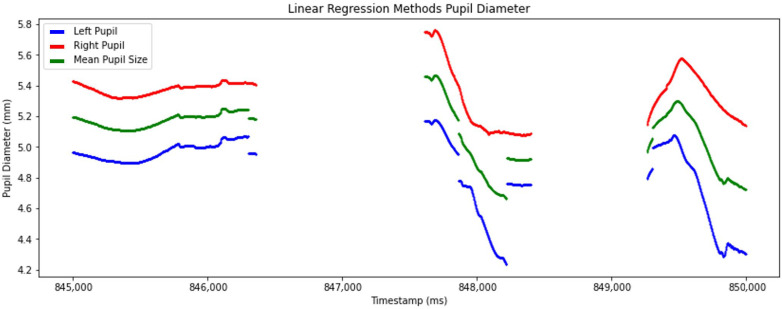
Detailed view of processed pupil diameter data after applying linear regression to predict the mean pupil diameter. The graph plots left pupil (blue line), right pupil (red line), and mean pupil diameter (green line) over timestamps.

Linear regression was applied in this study to forecast pupil diameter in one eye based on the measurements from the other eye [26]. This approach produced accurately mean pupil diameter computation (Figure 8).

**Figure 9:**
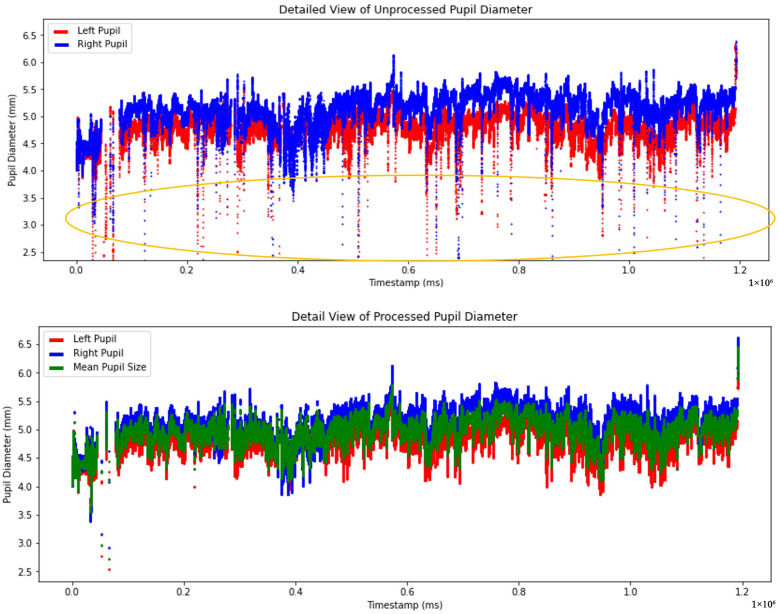
Comparison of unprocessed and processed pupil diameter data from timestamp 0 to the end time. The circled area highlighted noisy data in the unprocessed pupil.

Figure 9 demonstrates the difference between the raw and processed pupil diameter measurements. The top graph presents the unprocessed pupil diameter measurements with the left pupil represented in red and the right pupil in blue. The bottom graph presents the processed pupil diameter data, where artifacts, outliers, and missing values have been addressed. Additionally, the mean pupil diameter represented in green was calculated using a linear regression algorithm based on left and right pupil measurements. These approaches ensure a more accurate and robust representation of pupil size for subsequent analyses.

### 2.10. Calculation of Normalized Pupil Dilation Diameter

A baseline pupil diameter was initially determined to compute the normalized pupil dilation diameter. The original dataset was examined starting from the first timestamp to identify a period where both left and right eye pupil measurements were valid and recorded consecutively for a minimum of 400 milliseconds. This time frame was marked for further examination. The mean baseline pupil diameter was calculated based on the processed data within the identified period, ensuring that the dataset was refined before obtaining the final mean pupil diameter [27].

Once the baseline pupil diameter was obtained, the normalized pupil diameter was computed using the following formula [10]:

(7) $$Normalized\;Pupil\;Diameter\;=\;\frac{Pupil\;Diameter\;-\;Baseline\;Pupil\;Diameter}{Baseline\;Pupil\;Diameter}$$

Next, the mean normalized pupil diameter was calculated as the average of all normalized pupil diameter values.

**Figure 10:**
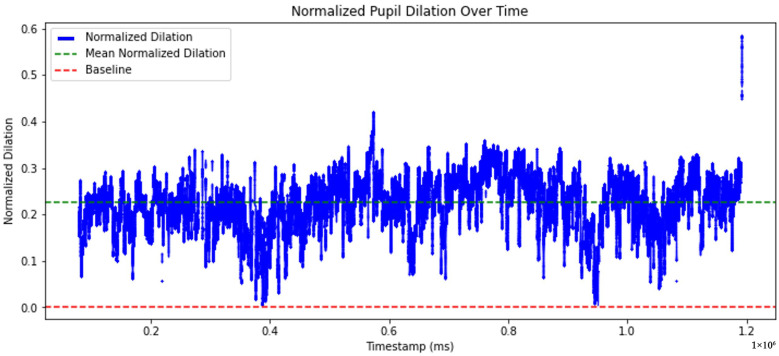
Time-series of normalized pupil dilation.

The graph shown in Figure 10 presents the normalized pupil dilation over time. The normalized pupil diameter is represented by the blue line indicating fluctuations in dilation over time. The green dashed line indicates the mean normalized dilation. The red dashed line represents the baseline, corresponding to a neutral reference level.

### 2.11. Integration of Multimodal Data- Full Outer Join

After preprocessing the pupil diameter data, the facial expression data was extracted from the raw multimodal dataset. To ensure consistency across datasets, the timestamps of the facial expression data were rounded to the nearest millisecond. As the facial expression data was recorded at 30 Hz, the upsampled pupil diameter data (1000 Hz) was subsequently downsampled to match the same temporal resolution. This step was critical to avoid inconsistencies in sampling rates and prevent timestamp misalignment between the two datasets.

**Figure 11:**
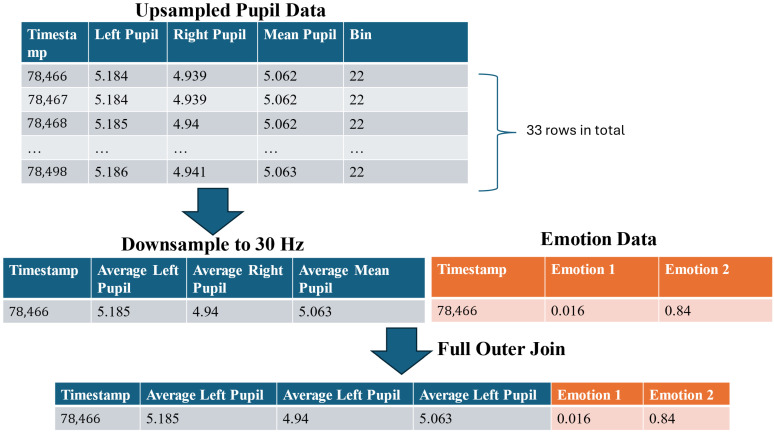
The workflow for integrating processed pupil diameter and facial expression data.

Downsampling was performed by dividing pupil diameter’s timestamp into bins corresponding to a 30 Hz sampling interval, which equates to 33 ms per bin. Given the 1000 Hz upsampling, each bin typically contained 33 data points. Within each bin, the average pupil diameters was calculated and used as the representative value for that interval. This approach preserves ensuring temporal alignment with facial expression data (Figure 11).

To integrate the two datasets, a full outer join was applied to merge all rows from the multimodal data. For this paper, this approach integrated the facial expression and pupil diameter data, preserving all time points from both datasets (Figure 11).

### 2.12. Software and Computational Environment

All data preprocessing was performed using Python (3.11.11). The code utilizes the following libraries: pandas (2.2.3), NumPy (2.2.4), SciPy (1.15.2), scikit-learn (1.6.1), and matplotlib (3.10.1). The raw data were exported from iMotions 10 (version 10.1.38911.4). Additional details are provided in the Supplementary Material (see README file).

## 3. Results

This study provided a generalized preprocessing pipeline for pupillometry signal analysis to process pupillometry data in multimodal research.

The key contributions of this paper include: (1) documented pipeline for pupillometry preprocessing. Our paper explicitly describes each stage of pupillometry preprocessing, including data cleaning, artifact removal, interpolation, outlier detection, normalization, and integration. This approach makes the process more accessible for researchers, including those new to multimodal pupillometry data analysis. (2) Multimodal data synchronization: there were challenges of timestamps mismatch among multimodal raw data due to sampling rate difference and after interpolation to 1000 Hz. To resolve this issue, timestamps were rounded to the nearest millisecond, ensuring consistency and facilitating seamless integration with facial expression response data. (3) Enhanced outlier detection: this study advances existing outlier detection methods by integrating a moving average algorithm with traditional techniques. The enhanced outlier detection method effectively identifies local outliers, while the original outlier primarily detected global outliers. By employing both the original and improved outlier detection methods, the study successfully removes both local and global outliers, resulting in a more comprehensive and accurate data. (4) Improved upsampling method: this study utilized the Piecewise Cubic Hermite Interpolation Polynomial (PCHIP) to upsampling the data to 1000 Hz. PCHIP demonstrated better performance compared to linear interpolation and cubic-spline interpolation methods. The PCHIP method maintains shape-preserving properties by ensuring monotonicity, preventing local overshooting of data points, and providing smooth transitions between adjoining data points to achieve more accurate upsampling data. (5) Handling missing pupil diameter data: this study introduced a linear regression algorithm to reconstruct the missing left or right pupil measurements to obtain the mean pupil diameter.

After applying the proposed preprocessing pipeline to the raw pupillometry data, we observed substantial improvements in data quality and reliability. Outliers and invalid data caused by blinks, head movements, or tracking errors were effectively eliminated. The data was also upsampled using PCHIP, resulting in smoother and more accurate reconstruction of missing data segments and outperforming linear interpolation. The processed pupillometry data was successfully integrated with facial expression metrics, facilitating seamless multimodal analysis.

## 4. Discussion

In this study, we present a series of methodological advancements aimed at improving the preprocessing of pupil diameter data within a multimodal platform. Our preprocessing pipeline builds upon some key steps described by Kret and Sjak Shie [11], whose method we reference in this manuscript. Specifically, we adopt their median comparison method to detect global outliers in the pupil diameter signal (Figure 5) [10]. To enhance this, we introduced a moving average-based algorithm that effectively identifies local outliers by capturing sudden spikes that deviate from local trends (Figure 4). This two-steps process ensures that both global and local anomalies are addressed, resulting in more precise data preprocessing.

For handling missing data, we applied PCHIP to interpolate missing data. This was adopted from Dan et al.’s (2020) paper, which is one of the best approaches to achieving the smallest Maximum Deviation [20], which preserves the monotonicity and shape of the pupil diameter signal, resulting in lower mean Root Mean Squared Error (RMSE) than traditional linear interpolation. Additionally, we leveraged linear regression to forecast pupil diameter in one eye based on the measurements from the other eye, further enhancing the robustness and completeness of our dataset [26].

Despite the effectiveness of the proposed preprocessing pipeline, certain limitations must be acknowledged. First, the study did not collect baseline pupil diameter measurements during data collection. Instead, a pre-event period was chosen as the baseline where both the left and right eye pupil measurements were valid and consecutive for at least 400 milliseconds. However, this approach may not accurately represent each participant’s true baseline pupil diameter, as it assumes that pupil diameter remains stable during this period. Pupil diameter can be impacted by cognitive load, environmental lighting, or spontaneous fluctuation. Future studies should include a controlled baseline measurement phase before experimental tasks to ensure more reliable baseline pupil diameter estimations.

Second, the study assumed a smooth progression of pupil diameter changes and relied on linear regression to handle missing data. While this technique is effective in many cases, it may introduce biases or fail to accurately reconstruct missing values when pupil diameter variations are rapid or irregular. To mitigate this limitation, more advanced imputation techniques, such as machine learning-based methods, might be useful in future research to improve the accuracy of missing data estimation.

Third, consistent pupil dilation speeds are assumed by the MAD-based outlier detection, which may remove meaningful fluctuations caused by cognitive or physiological changes. To reduce this risk, alternative anomaly detection methods, such as adaptive thresholding or deep learning-based approaches could be explored to distinguish genuine variations from artifacts more effectively.

Fourth, Dan et al. (2020) recommended three interpolation methods for pupil diameter data. However, their study only compared six interpolation methods [20]. Future research could expand this comparison by incorporating additional interpolation techniques or leveraging machine learning approaches to enhance interpolation accuracy and adaptively select the most suitable method for different pupil response patterns.

Fifth, while the preprocessing pipeline improves data quality and reliability, it does not fully eliminate noise from outside sources, such as lighting conditions or head movement. Future studies should consider integrating additional noise reduction strategies, such as adaptive filtering techniques, different interpolation methods, or hardware improvements, to further enhance data integrity. In the future, a software platform could be potentially built to allow automated processing of multimodal data.

Finally, while the pipeline is demonstrated using data from a single participant for clarity and focusing on explaining the preprocessing methods in detail, it has been successfully applied to all 25 participants in the datasets. This dataset was chosen as the input data because endoscopic procedures require high cognitive load and visual attention demands, making pupil responses and facial expressions particularly relevant for analysis. The preprocessing pipeline is robust and generalizable, applicable to multimodal pupil diameter analysis across diverse research domains. Furthermore, the methodology is not specific to the iMotions system; all algorithms require only standard pupil diameter and timestamp data, which are available from most eye-tracking platforms. The approach also incorporates robust time series integration and data alignment techniques, supporting the combination of eye-tracking data with other modalities and making it well-suited for multimodal research and a wide range of experimental setups. Furthermore, this pipeline is currently being utilized in our separate project on Eye-Hand Coordination in Central Line Placement, where it has been effectively applied to data from 12 participants. In this project, we have integrated hand tracking data alongside eye tracking, enabling comprehensive analysis of eye-hand coordination during central line procedures. This demonstrates the flexibility and adaptability of the methodology across different experimental contexts and participant groups.

## 5. Conclusions

In conclusion, the approach we presented effectively addressed challenges such as timestamp misalignment, outlier detection, missing data interpolation, and multimodal integration through robust filtering techniques, data alignment strategies, and interpolation techniques. These methods enhance dataset accuracy and reduce biases associated with missing values, thereby increasing the reliability of the dataset for further analyses compared to traditional techniques. Compared to existing pupillometry studies, our approach enhances robustness and synchronization with facial expression data, enabling more precise multimodal analyses of cognitive and emotional responses during complex tasks. To our knowledge, this is the first study to present a robust and systematic preprocessing pipeline for pupil diameter data obtained from a multimodal platform across multiple participants and experimental contexts, advancing the reliability and reproducibility of pupillometry preprocessing in cognitive and clinical research domains. Overall, the proposed approach offers a systematic approach for handling multimodal pupillometry data, ensuring accurate results while keeping important information for further analysis.

## Figures and Tables

**Table 1:** Raw pupil diameter and facial expression measurements.

Timestamp	Left Pupil	Right Pupil	Facial Expression1	Facial Expression 2
63,212	Nan	Nan	27.53	0.31
63,216.17	5.150	5.023	Nan	Nan
63,257.62	5.148	5.02	Nan	Nan

## Data Availability

The dataset was collected from an endoscopy novice performing a VR simulation using iMotion 10. The data is included in the document. The source code is available in Python on GitHub (version 3.17.2) at https://github.com/DavidOng122/multimodal-pupii-preprocessing (accessed on 27 July 2025).

## Supplementary Materials

The following supporting information can be downloaded at: https://www.mdpi.com/article/10.3390/s25154737/s1, Figure S1: Left Eye Pupil Interpolated Using Mikima Method; Figure S2: Left Eye Pupil Interpolated Using PCHIP Method; Figure S3: Left Eye Pupil Interpolated Using Akima Method; Pupil_Preprocessing_Functions.py: Python functions for cleaning, filtering, interpolating normalizing, and integrating pupil diameter measurements. Example.py: Example script demonstrating the usage of functions in Pupil_Preprocessing_Functions.py. README.txt: Instrctions and guidance on how to use the provided code files. Requirements.txt: Lists the Python libraries required to run the provided code.

## Abbreviations

The following abbreviations are used in this manuscript:

MAD	Moving Absolute Deviation
MA	Moving Average
MM	Millimeter
MS	Millisecond
PCHIP	Piecewise Cubic Hermite Interpolating polynomial
VR	Virtual Reality
