# Supplementary figures and images for: A Preprocessing Pipeline for Pupillometry Signal from Multimodal iMotion Data
Source: Sensors (Basel). 2025 Jul 31;25(15):4737. doi: 10.3390/s25154737 (PMC12349379; doi:10.3390/s25154737)

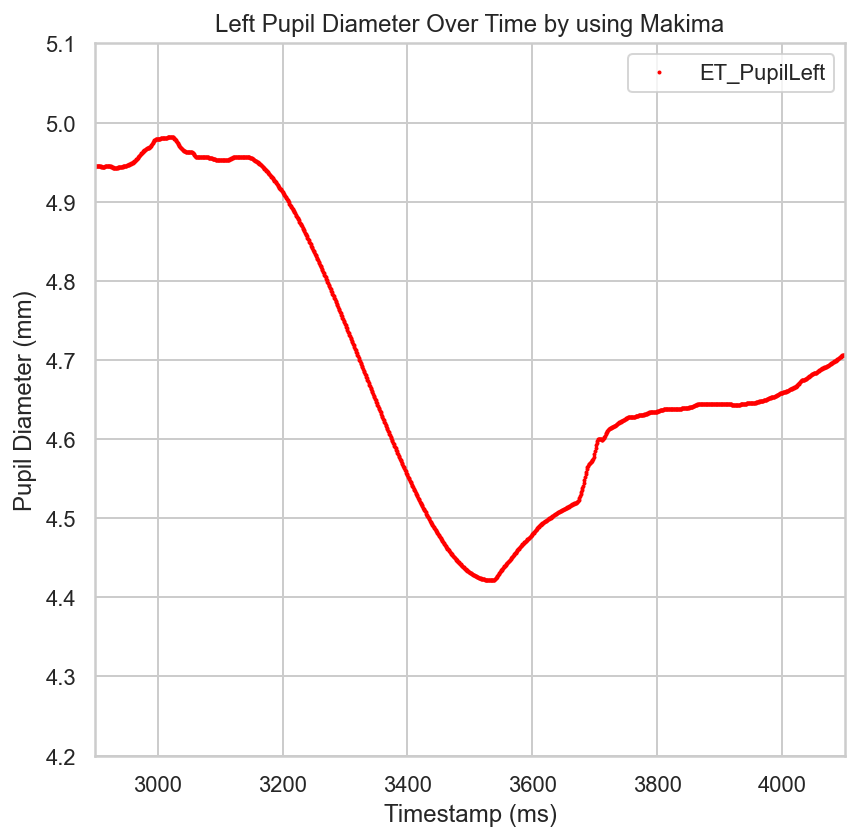

Supplement: Supplementary file 1 [file sensors-25-04737-s001.zip › Supplementary files/pupil data in different interpolation method/Figure S1.png]

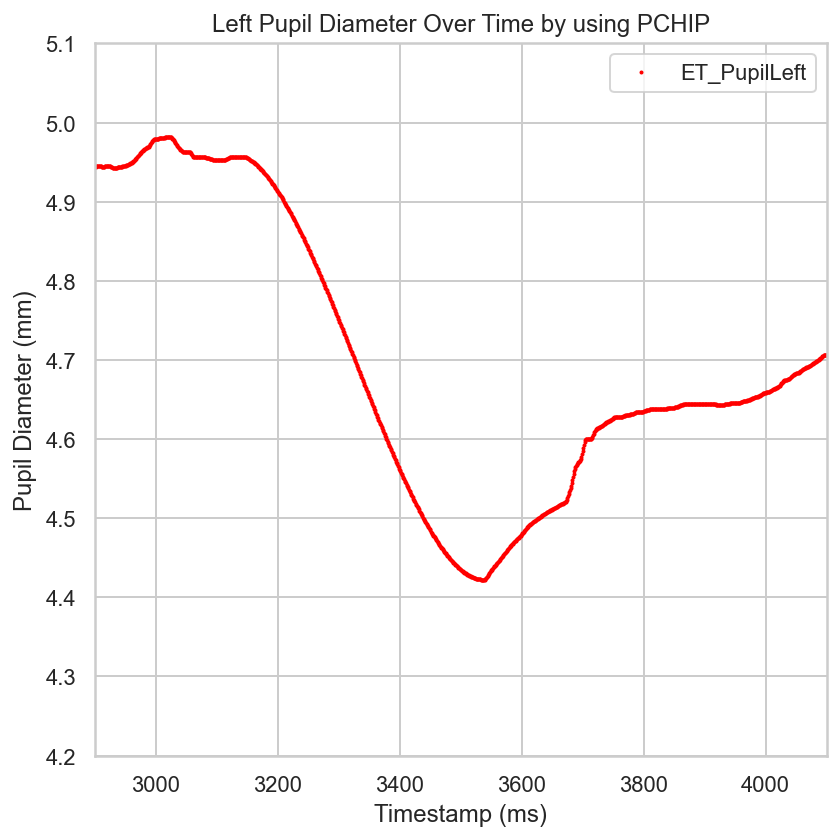

Supplement: Supplementary file 1 [file sensors-25-04737-s001.zip › Supplementary files/pupil data in different interpolation method/Figure S2.png]

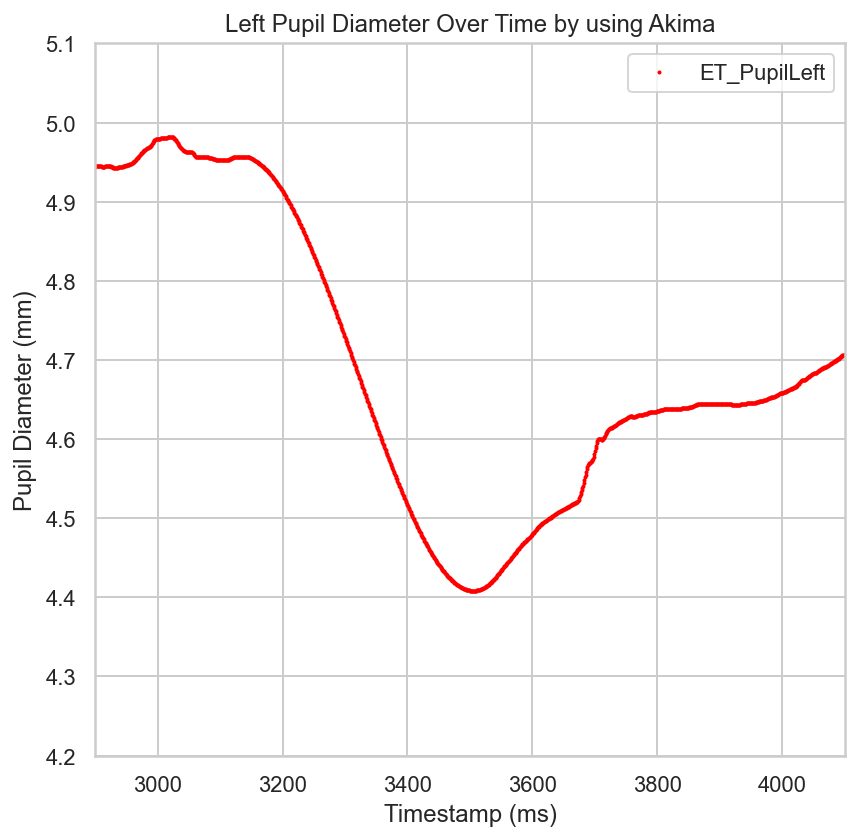

Supplement: Supplementary file 1 [file sensors-25-04737-s001.zip › Supplementary files/pupil data in different interpolation method/Figure S3.png]
